# Phenotypes controlled by the *Brucella abortus* two component system BvrR/BvrS are differentially impacted by BvrR phosphorylation

**DOI:** 10.3389/fmicb.2023.1148233

**Published:** 2023-05-10

**Authors:** Pamela Altamirano-Silva, Jazmín Meza-Torres, Ana Mariel Zúñiga-Pereira, Sigrid Zamora-Jaen, Natalia Pietrosemoli, Gabriela Cantos, Johann Peltier, Javier Pizarro-Cerdá, Edgardo Moreno, Carlos Chacón-Díaz, Caterina Guzmán-Verri, Esteban Chaves-Olarte

**Affiliations:** ^1^Centro de Investigación en Enfermedades Tropicales, Facultad de Microbiología, Universidad de Costa Rica, San José, Costa Rica; ^2^Bioinformatics and Biostatistics Hub, CNRS USR3756, Institut Pasteur, Université Paris Cité, Paris, France; ^3^Laboratoire Pathogenèse des Bactéries Anaérobies, CNRS UMR6047, Institut Pasteur, Université Paris Cité, Paris, France; ^4^CEA, CNRS, Institute for Integrative Biology of the Cell (I2BC), Université Paris-Saclay, Gif-sur-Yvette, France; ^5^Yersinia Research Unit, CNRS UMR6047, Institut Pasteur, Université Paris Cité, Paris, France; ^6^Programa de Investigación en Enfermedades Tropicales, Escuela de Medicina Veterinaria, Universidad Nacional, Heredia, Costa Rica

**Keywords:** two-component system, phosphorylation, response regulator, virulence, pattern, *Brucella*, intracellular survival

## Abstract

*Brucella abortus* is a zoonotic pathogen whose virulence depends on its ability to survive intracellularly at the endoplasmic reticulum derived compartment. The two-component system BvrR/BvrS (BvrRS) is essential for intracellular survival due to the transcriptional control of the type IV secretion system VirB and its transcriptional regulator VjbR. It is a master regulator of several traits including membrane homeostasis by controlling gene expression of membrane components, such as Omp25. BvrR phosphorylation is related to DNA binding at target regions, thereby repressing or activating gene transcription. To understand the role of BvrR phosphorylation we generated dominant positive and negative versions of this response regulator, mimicking phosphorylated and non-phosphorylated BvrR states and, in addition to the wild-type version, these variants were introduced in a BvrR negative background. We then characterized BvrRS-controlled phenotypes and assessed the expression of proteins regulated by the system. We found two regulatory patterns exerted by BvrR. The first pattern was represented by resistance to polymyxin and expression of Omp25 (membrane conformation) which were restored to normal levels by the dominant positive and the wild-type version, but not the dominant negative BvrR. The second pattern was represented by intracellular survival and expression of VjbR and VirB (virulence) which were, again, complemented by the wild-type and the dominant positive variants of BvrR but were also significantly restored by complementation with the dominant negative BvrR. These results indicate a differential transcriptional response of the genes controlled to the phosphorylation status of BvrR and suggest that unphosphorylated BvrR binds and impacts the expression of a subset of genes. We confirmed this hypothesis by showing that the dominant negative BvrR did not interact with the *omp25* promoter whereas it could interact with *vjbR* promoter. Furthermore, a global transcriptional analysis revealed that a subset of genes responds to the presence of the dominant negative BvrR. Thus, BvrR possesses diverse strategies to exert transcriptional control on the genes it regulates and, consequently, impacting on the phenotypes controlled by this response regulator.

## 1. Introduction

*Brucella abortus* is an *Alphaproteobacteria* facultative extracellular-intracellular pathogen responsible for brucellosis, one of the most distributed bacterial zoonoses in the world (Moreno, 2014). This bacterium causes economic losses due to abortion in cattle and is of public health concern since it produces a debilitating febrile disease in humans by ingestion of unpasteurized products or occupational exposure (Moreno and Moriyon, 2006; McDermott et al., 2013). The pathogenesis of brucellosis is intimately linked to the ability of *B. abortus* to enter eukaryotic cells modulating the intracellular trafficking to reach the endoplasmic reticulum (ER). Within this organelle, the bacterium enters a multiplication phase essential for disseminating throughout the infected host (Celli, 2015, 2019).

Several molecular determinants are essential for the intracellular lifestyle of *B. abortus*. Among those stands the two-component system (TCS) BvrR/BvrS (BvrRS) which has been consistently identified in independent unbiased approaches designed to understand the virulence mechanisms exerted by *B. abortus* (López-Goñi et al., 2002; Viadas et al., 2010; Altamirano-Silva et al., 2018). BvrRS is part of an operon encompassing 16 genes, with orthologs in the phylogenetically related endosymbiont *Sinorhizobium meliloti*, and the plant pathogen *Agrobacterium tumefaciens* (Rivas-Solano et al., 2022). It is composed of the sensor protein BvrS, which, upon activation, is auto-phosphorylated and then transfers the phosphate moiety to aspartate 58 of the response regulator, BvrR (Altamirano-Silva et al., 2018). BvrR phosphorylation induces a conformational change that increases the protein’s affinity to promoter regions, impacting gene expression (Sola-Landa et al., 1998; Altamirano-Silva et al., 2018; Rivas-Solano et al., 2022).

The TCS BvrR/BvrS controls several phenotypes related to the intracellular life cycle of *B. abortus*. *B. abortus* activates small Rho GTPases during the entry process to nonprofessional phagocytic cells. BvrRS-defective mutants lack this ability indicating that this TCS controls the expression of yet unknown, molecules that participate in this process (Sola-Landa et al., 1998; Guzmán-Verri et al., 2001). Once inside cell, the BvrR phosphorylation is triggered by low pH and nutrient limitation, inducing the expression of the transcriptional regulator VjbR and the Type IV Secretion System VirB (Altamirano-Silva et al., 2018). Assembly of the latter is crucial to inject protein effectors that allow *B. abortus* to evade the lysosomal route, reaching the ER (Celli et al., 2003; Myeni et al., 2013). At late stages of infection, BvrRS activates again the expression of both VjbR and VirB to allow bacterial egress and interaction with new host cells (Altamirano-Silva et al., 2021).

BvrRS activity also has a profound impact on membrane homeostasis. BvrRS exerts transcriptional control on membrane proteins such as Omp3a (Omp25) and Omp3b and enzymes related to LPS synthesis (Guzman-Verri et al., 2002; Rivas-Solano et al., 2022). BvrRS-defective mutants show abnormal LPS acylation patterns and are susceptible to cationic peptides such as polymyxin B (Manterola et al., 2005, 2007). Direct binding of phosphorylated BvrR to regulatory regions of tamA*, pckA*, *omp25, virB1* and its own regulatory region has been shown (Rivas-Solano et al., 2022). Overall, BvrRS influences the expression of more than 100 genes (Viadas et al., 2010). Some of these genes encode enzymes at the metabolic crossroads of carbon and nitrogen pathways, reinforcing the role that BvrRS has in the coordination of gene expression, required for a successful *B. abortus* infection (Rivas-Solano et al., 2022), a phenomenon also reflected in the proteome of the *bvrR* and *bvrS* mutants compared to wild-type *Brucella* (Lamontagne et al., 2007).

Despite the profound transcriptional impact of BvrRS and its role in controlling several crucial phenotypes, little is known about the molecular mechanisms used by this system to regulate such a diverse variety of genes. In this work, we generated transcriptional regulator BvrR dominant positive and negative mutants mimicking phosphorylated and non-phosphorylated BvrR states, that were introduced, in addition to the wild-type version, in a BvrR*-*negative bacterial background. Analysis of the phenotypes and transcriptional responses in the panel of strains generated indicates the existence of diverse strategies used by BvrR to control transcriptional responses and phenotypes.

## 2. Materials and methods

### 2.1. Bacterial strains and growth conditions

The bacterial strains listed in Table 1 were grown *in vitro* at 37°C in tryptic soy broth (TSB) for 24 h to stationary phase, and aliquots were frozen at −70°C in TSB-glycerol 20%. A frozen stock of brucellae was thawed 48 h before the assays. Then, bacteria were grown in 20 mL of TSB in glass flasks at 200 rpm and 37°C for 18 h. The optical density was measured at 420 nm (OD_420_). The bacterial population was estimated by plotting the OD_420_ on a standard curve, and 5 × 10^9^ bacteria were inoculated in 20 mL of TSB and incubated with agitation at 200 rpm and 37°C. When needed, *B. abortus* strains were supplemented with antibiotics to maintain plasmid selection. Aliquots were taken at the indicated times for bacterial growth determination, by optical density measurement at OD_420_. For the assays where bacteria were used at the mid-exponential growth phase (OD_420_ = 0.3–0.5), 5 × 10^9^ bacteria were inoculated in 20 mL of TSB and incubated with agitation at 200 rpm and 37°C for 16 h.

**Table 1:** Strains used in this study.

*Brucella* strains	Characteristics or relevant features	Source of references
*B. abortus* 2308 W (Ba_2308_)	WT *Brucella* strain, virulent, smooth LPS; NaI^r^	Sangari and Agüero (1991)
*B. abortus bvrR*-	Smooth-LPS strain derived from *B. abortus* 2308 NaI^r^ with a mini-Tn5 insertion in the bvrR gene	Sola-Landa et al. (1998)
*B. abortus bvrR*- empty vector (Ba_R-_)	Smooth-LPS strain derived from *B. abortus* 2308 NaI^r^ with a mini-Tn5 insertion in the *bvrR* genes and harboring the plasmid prH002	Altamirano-Silva et al. (2018)
*B. abortus bvrR*-p*bvrR* (Ba_R-_R)	*B. abortus bvrR-* mutant harboring the plasmid prH002*bvrR* with a 738-bp insert corresponding to bvrR; Cm^r^ BaR-R = complemented/rescue strain	Altamirano-Silva et al. (2018)
*B. abortus bvrR*-p*bvrR* D58E (Ba_R-_RD58E)	*B. abortus bvrR*-negative mutant harboring the plasmid prH002*bvrR*D58E with a 738-bp insert corresponding to *bvrR*D58E; Cm^r^ BaR-RD58E = dominant-positive strain (D58E)	Altamirano-Silva et al. (2018)
*B. abortus bvrR*- p*bvrR* D58A (Ba_R-_RD58A)	*B. abortus bvrR*- mutant harboring the plasmid prH002*bvrR*D58A with a 738-bp insert corresponding to *bvrR*D58A; Cm^r^ BaR-RD58A = dominant-negative strain (D58A)	Altamirano-Silva et al. (2018)

### 2.2. Polymyxin B susceptibility assays

Stock solutions of polymyxin B were prepared in sterile 1 mM 4-(2-hydroxyethyl)-1-piperazineethanesulfonic acid (HEPES) pH 8.0. Serial dilutions of polymyxin were made in 96-well microtiter-type plates. 2 × 10^4^ CFUmL-1 of Ba_2308_, Ba_R-_, Ba_R-_RD58E, Ba_R-_R, and Ba_R-_RD58A grown to exponential growth phase were centrifuged and resuspended in TSB. Then, 100 μL of bacterial suspension were dispensed into the wells. Bacteria incubated without polymyxin were used as growth control. Plates were incubated for 1 h at 37°C, and 10 μL of serial dilutions of each well were inoculated onto tryptic soy agar (TSA) plates. The results were expressed as the percentage of survival.

### 2.3. Intracellular replication experiments

HeLa epithelial cells (ATCC clone CCL-2) were cultivated and infected with Ba_2308_, Ba_R-_, Ba_R-_RD58E, Ba_R-_R, and Ba_R-_RD58A as previously described (Altamirano-Silva et al., 2021). The cells were seeded in 24-well tissue culture plates 2 days before infection to obtain a final concentration of 5 × 10^5^ cells per well, and multiplicities of infection (MOI) of 500 were used. MOIs were adjusted based on the OD_420_ of the inoculum by diluting bacteria in Dulbecco’s Modified Eagle Medium (DMEM). Cells were infected with *B. abortus* strains, centrifuged for 5 min at 330× *g* at 4°C, incubated for 45 min at 37°C under 5% CO2, and washed with phosphate-buffered saline (PBS). Extracellular bacteria were eliminated by treatment with gentamicin at 100 μg/mL for 1 h, and cells were incubated for the indicated times in the presence of gentamicin at 5 μg/mL.

### 2.4. Intracellular replication quantitation

The number of intracellular viable *B. abortus* strains was determined at different h post- infection. Cells were washed twice with PBS and treated for 10 min with Triton X-100 (0.1%). Lysates were serially diluted, plated on tryptic soy agar dishes, and incubated at 37°C for 3 days under 5% CO_2_ to quantify colony-forming units (CFU).

### 2.5. Immunofluorescence microscopy

HeLa epithelial cells were seeded in 24-well tissue culture plates on 12-mm-diameter glass coverslips 2 days before infection to obtain a final concentration of 5 × 10^5^ cells per well and infected with Ba_2308_, Ba_R-_, Ba_R-_RD58E, Ba_R-_R, and Ba_R-_RD58A at a MOI of 500 as described above. At 48 h post-infection, coverslips were washed three times in 1X PBS and fixed using 3% paraformaldehyde in PBS (pH 7.4) at 37°C for 10 min. Rabbit anti-calnexin polyclonal antibody ab75801 (Abcam) was used to localize intracellular compartments. As reported elsewhere, in house polyclonal mouse antibodies to *B. abortus* were used to detect *Brucella* (Altamirano-Silva et al., 2021). An Alexa Fluor 488-conjugated goat anti-mouse antibody and an Alexa Fluor 594-conjugated anti-rabbit antibody (Life Technologies) were used as developing antibodies. Confocal analysis was performed with an Olympus U-TB190 (100X) under oil immersion. Confocal images of 1,024 by 1,024 pixels were acquired with the FV10-AV ver.03.01 software (Olympus) and assembled with Adobe Photoshop CS3 (Adobe Systems, San Jose, CA).

### 2.6. Electrophoretic and immunochemical analysis

Ba_2308_, Ba_R-_, Ba_R-_RD58E, Ba_R-_R, and Ba_R-_RD58A were grown in TSB at 37°C. At the mid-exponential growth phase, an aliquot of bacteria was taken and concentrated by centrifugation at 10,000× *g* for 10 min, resuspended in Laemmli sample buffer, and heated at 100°C for 20 min. According to the manufacturer’s instructions, the protein concentration was determined by the Bio-Rad DC method, and equal amounts of protein (20 μg) were loaded onto a 12.5% gel for SDS-PAGE. Separated proteins were transferred to a polyvinylidene difluoride (PVDF) membrane and probed with the indicated antibodies. Rabbit-anti VirB8 antibodies, anti-VjbR and anti-BvrR were produced by immunization with recombinant proteins followed by affinity chromatography purification as previously described (Martínez-Núñez et al., 2010). Mouse monoclonal anti-Omp25 and anti-Omp19 antibodies were kindly provided by Dr. Axel Cloeckaert. Membranes were further incubated with peroxidase-conjugated anti-mouse (Invitrogen cat#G21040) or anti-rabbit antibodies (Invitrogen cat#G21234), and a chemiluminescence reaction visualized the detected bands.

### 2.7. Chromatin immunoprecipitation assay

Ba_2308_, Ba_R-_, Ba_R-_RD58E, Ba_R-_R, and Ba_R-_RD58A were grown in TSB at 37°C to mid-exponential growth phase. Bacteria were fixed with 1% formaldehyde and incubated at room temperature for 10 min. Then, the reaction was quenched with 125 mM glycine for 5 min. Bacteria were washed with cold phosphate-buffered saline twice and the cells were lysed in 0.6 mL of lysis solution (10 mM Tris pH 8.0, 50 mM NaCl, 10 mM EDTA, 20% sucrose, 20 mg mL-1 lysozyme) and 0.6 mL of 2X RIPA solution (100 mM Tris pH 8.0, 300 mM NaCl, 2% Igepal, 1% sodium deoxycholate, 0.2% SDS). To fragment DNA in an average size of 500 bp, the cell extracts were sonicated and centrifuged for 30 min at 10,000× *g*. Supernatants were stored at −80°C. An aliquot of the extract was taken as a control of total DNA before immunoprecipitation and referred to as total DNA.

For immunoprecipitation, 100 μL of 10 mg/mL SureBeads^™^ Protein A Magnetic Beads were incubated with 2 μg of BvrR antibodies for 1 h at room temperature. Then, bacterial lysates were incubated with the preloaded beads for 1 h at room temperature. The beads were washed three times with 1X RIPA solution. The immunoprecipitated material was eluted with 30 μL of 20 mM glycine pH 2.0 for 5 min at room temperature and neutralized with 80 μL of elution buffer (25 mM Tris pH 8.0, 5 mM EDTA, 0.5% SDS). Cross-linking of the immunoprecipitated was reversed by incubation at 65°C overnight. The immunoprecipitated and the total DNA were purified using the DNeasy^®^ Blood & Tissue Kit (Qiagen) according to the manufacturer’s instructions. P*_omp25_*, and P*_vjbR_*, were amplified by quantitative real-time PCR an Applied Biosystems StepOnePlus^™^ Real-Time PCR instrument using primers P*_omp25_*F (5′-CCGCAATTACCCTCGATATGT-3′) and P*_omp25_*R (5′-ATGGCATTCTCCTTACACAAATTAC-3′) and P*_vjbR_*F (5′- TAAGCGATTGAAGGCCTC-3′) and P*_vjbR_*R (5′- CTCATTGGAAATATCCTTGGTGAT-3′), respectively. The standard curve method was used for relative quantification. Data were presented as a percentage of precipitated DNA (IP)/total DNA (IN) (Uzureau et al., 2010).

### 2.8. RNA isolation and sequencing

Ba_2308_, Ba_R-_, Ba_R-_RD58E, Ba_R-_R, and Ba_R-_RD58A were grown in TSB to exponential growth phase and centrifuged at 5,000× *g* for 5 min at 4°C. Samples were resuspended in 250 μL of TE buffer, pH 8.0 with 1 mg/mL lysozyme, and incubated for 5 min at room temperature. RNA was then isolated using the RNeasy Midi Kit (Qiagen) with a DNase treatment. RNA was eluted from the column using RNase-free water. Total RNA concentration was calculated by Quant-IT RiboGreen (Invitrogen). To assess the integrity of the total RNA, samples were run on the TapeStation RNA screen tape (Agilent). High-quality RNA preparations with RIN higher than 7.0 were used for RNA library construction. The library was prepared with 1 μg of total RNA for each sample by Illumina TruSeq mRNA Sample Prep kit (Illumina, Inc., San Diego, CA, United States). Briefly, bacterial rRNA was depleted using the NEBNext rRNA Depletion Kit (Bacteria) (NEB). After depleting the rRNA, the remaining RNA was fragmented using divalent cations under elevated temperature. Synthesis of cDNA from the cleaved RNA fragments was performed using SuperScript II reverse transcriptase (Invitrogen) and random primers. This was followed by double-strand cDNA synthesis using DNA Polymerase I and RNase H. These cDNA fragments then went through an end repair process, the addition of a single ‘A’ base, and the ligation of the indexing adapters. The products were then purified and enriched with PCR to create the final cDNA library. The libraries were quantified using KAPA Library Quantification Kits for Illumina Sequencing platforms according to the qPCR Quantification Protocol Guide (KAPA) and qualified using the TapeStation D1000 ScreenTape (Agilent). Indexed libraries were then submitted to an Illumina NovaSeq (Illumina, Inc., San Diego, CA, United States), and the paired-end (2 × 151 bp) (~40 M reads per sample ~6G/sample) sequencing was performed by Macrogen Incorporated (South Korea).

### 2.9. RNA-seq mapping and quantification

After trimming the adaptors (cutadapt, v. 2.4) (Martin, 2011), high-quality reads averaging 28.4 million reads per sample were obtained. The resulting count matrix of raw data and the experimental design are available at:10.5281/zenodo.7548947. Sequencing quality was assessed for each sample (before and after mapping) using MultiQC (v. 1.6) (Ewels et al., 2016). Reads were aligned on the *B. abortus genome* (*Brucella abortus* strain Wisconsin) (Suárez-Esquivel et al., 2016) from GenBank (Clark et al., 2016) using bowtie2 (v. 2.3.4.1) (Langmead and Salzberg, 2012). The reference genome annotation was complemented with NCBI Reference Sequence GenBank: LT671512.1 (Chormosome 1) and NZ_LT671513.1 (Chromosome 2). Read summarization and annotations were done with the featureCounts (v 1.6.14), requiring both read ends to map. This tool was run twice to identify genes and pseudo-genes features on the genome. Functional annotation of the genes/pseudogenes gene ontologies was obtained using the annotations provided by PATRIC (Davis et al., 2020). The resulting genome annotation was manually curated using an in-house annotation.

### 2.10. RNA-seq statistical analysis

Gene expression profiles were analyzed using the R software (R: A Language and Environment for Statistical Computing, 2017) and several Bioconductor (Gentleman et al., 2004) packages, including DESeq2 (Love et al., 2014) (v 1.26.0) and SARTools (v 1.7.0) (Varet et al., 2016). The statistical analysis included (i) data description, (ii) data normalization and exploration (iii) testing for differential expression for each gene/pseudogene between the strains.

- Dataset description: The dataset included 24 samples from 5 different strains, each having the number of replicates indicated in parenthesis Ba_R-_R (5), *Ba_2308_*, (5), Ba_R-_RD58A (5), Ba_R-_RD58E (5), and *Ba*_R-_(4). For this dataset, 3,301 genes/pseudogenes were identified for each condition.
- Data normalization and exploration: Data normalization was performed according to the DESeq2 model and package. Normalized read counts were obtained by dividing raw read counts by the scaling factor associated with the sample they belong to (locfunc = “median”). The data variability was explored by performing hierarchical clustering and principal component analysis (PCA) of the whole sample set after the counts were transformed using a variance stabilizing transformation. Hierarchical clustering was calculated using Euclidian distance and the Ward criterion for agglomeration. PCA was performed using DESeq2 (Love et al., 2014) (v 1.26.0).
- Differential analysis was performed to identify genes/pseudogenes having a significantly different expression between each pair of the different strains *Ba_2308_, Ba_R-_*, Ba_R-_RD58E, Ba_R-_R, and Ba_R-_RD58A using the R software (v. 1.2.5033), R packages DESeq2 (v 1.26.0) and SARTools (v 1.7.0) (Varet et al., 2016). The strategy was to fit one linear model per gene/pseudogene using a design of one factor (strain) to estimate the coefficients (log_2_FC) and corresponding value of *p*. Raw *p*-values were corrected for multiple testing using Benjamini and Hochberg’s method (Benjamini and Hochberg, 1995). Genes /pseudogenes having an adjusted *p* value <0.05 were considered differentially expressed for the given pairwise comparison. Heatmaps were generated with R (package pheatmap v 1.0.12), using genes with differential expression (|log2FC| > = 3.5) for at least one pairwise comparison.

## 3. Results

### 3.1. The phenotypes controlled by BvrRS are differentially impacted by the phosphorylation of BvrR

To analyze the impact of BvrR phosphorylation on the phenotypes controlled by the BvrRS, we took advantage of previously described mutations in the phosphorylated aspartate of response regulators that confer either dominant-negative (D58A) or dominant-positive phenotypes (D58E) (Klose et al., 1993). Plasmids containing the BvrR variations, and the wild-type isoform were introduced in a *bvrR- B. abortus* strain (Ba_R-_) to generate strains Ba_R-_RD58A, Ba_R-_RD58E and Ba_R-_R, respectively. Wild type *B. abortus* 2308 (Ba_2308_) and the derivative *bvrR*- strain complemented with an empty vector Ba_R-_ were used as positive and negative controls in all the phenotypic analyses.

**Figure 1:**
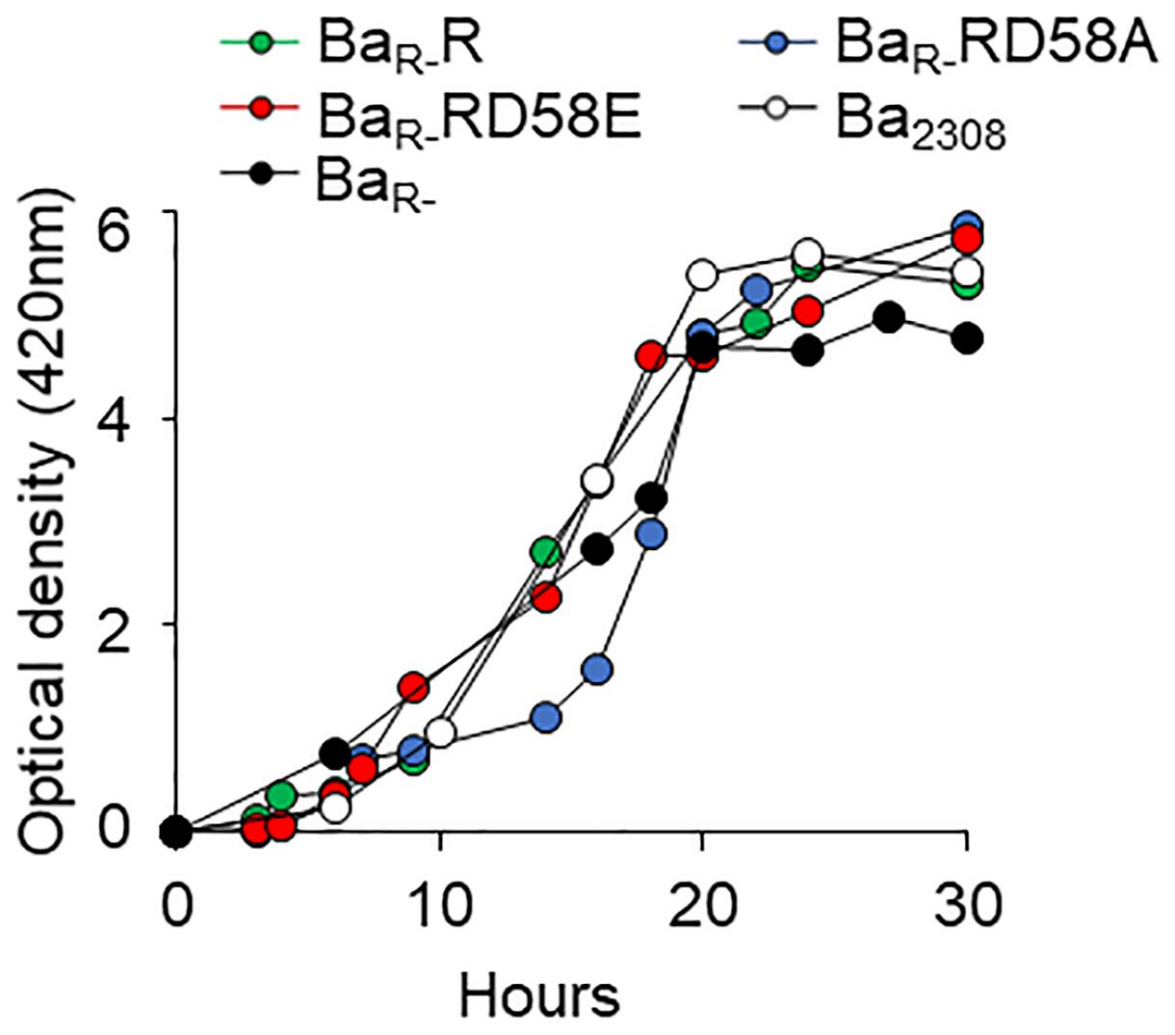
Effect of BvrR phosphorylation on growth kinetics of *B. abortus*. Ba_2308_ (white circles), Ba_R-_ (black circles), Ba_R-_RD58E (red circles), Ba_R-_R (green circles), and Ba_R-_RD58A (blue circles) were inoculated in TSB at 5 × 10^9^ CFU, and the optical density was measured at 420 nm over time. Each point represents the mean value of triplicates samples. Standard deviation in all cases was less than 1%. Representative curves from three independent experiments are shown.

To determine the effect of the BvrR phosphorylation on *in vitro* growth, strains were grown in tryptic soy broth (TSB) and the absorbance was measured at different times. All the strains followed a similar dynamic reaching the stages of the growth curve at approximately the same times but with minor delays of some strains, for instance Ba_R-_RD58A (Figure 1). Thus, the differences in phenotypes and gene expression reported below are not due to major differences in the ability to grow *in vitro*.

**Figure 2:**
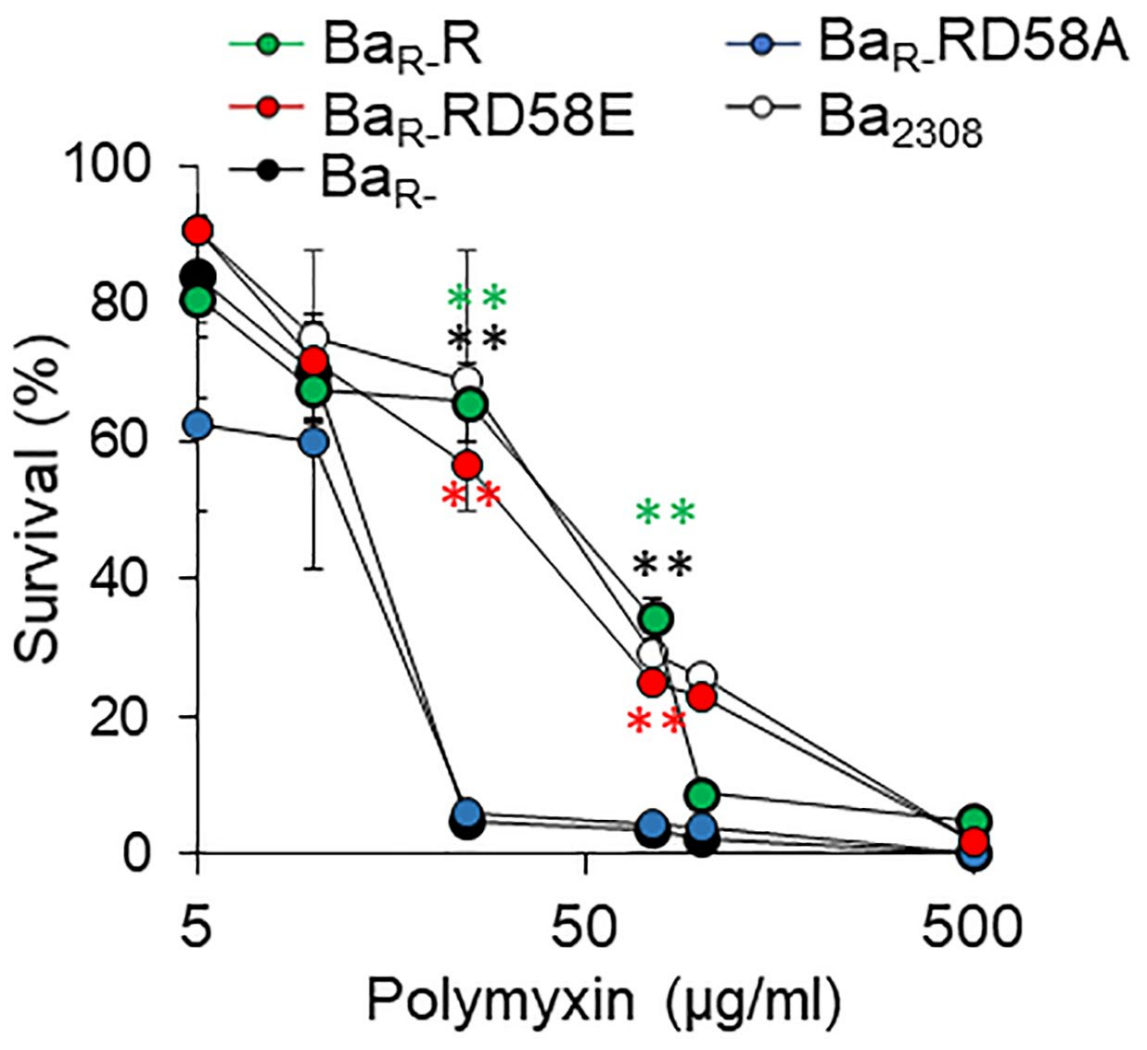
Effect of BvrR phosphorylation on polymyxin resistance of *B. abortus*. Ba_2308_ (white circles), Ba_R-_ (black circles), Ba_R-_RD58E (red circles), Ba_R-_R (green circles), and Ba_R-_RD58A (blue circles) grown to mid-exponential growth phase were exposed to increasing concentrations of polymyxin B for 60 min at 37°C. Then, bacterial viability was determined by plating on TSA plates. Means and standard deviations are shown; *n* = 3 independent experiments. Statistical significance was calculated by ANOVA and Tukey’s multiple-comparison test (**p < 0.005 compared to Ba_R-_).

To determine the effect of the BvrR phosphorylation on *B. abortus* resistance to cationic peptides we incubated the strains with different concentrations of polymyxin and determined their survival. As expected, Ba_R-_ was highly susceptible to polymyxin at 25, 50, and 75 μg/mL compared to Ba_2308_ (Figure 2). Ba_R-_RD58A was also highly susceptible to polymyxin B at 25, 50 and 75 μg/mL. In contrast, Ba_R-_R and Ba_R-_RD58E were as resistant to polymyxin B as Ba_2308_ (Figure 2).

**Figure 3:**
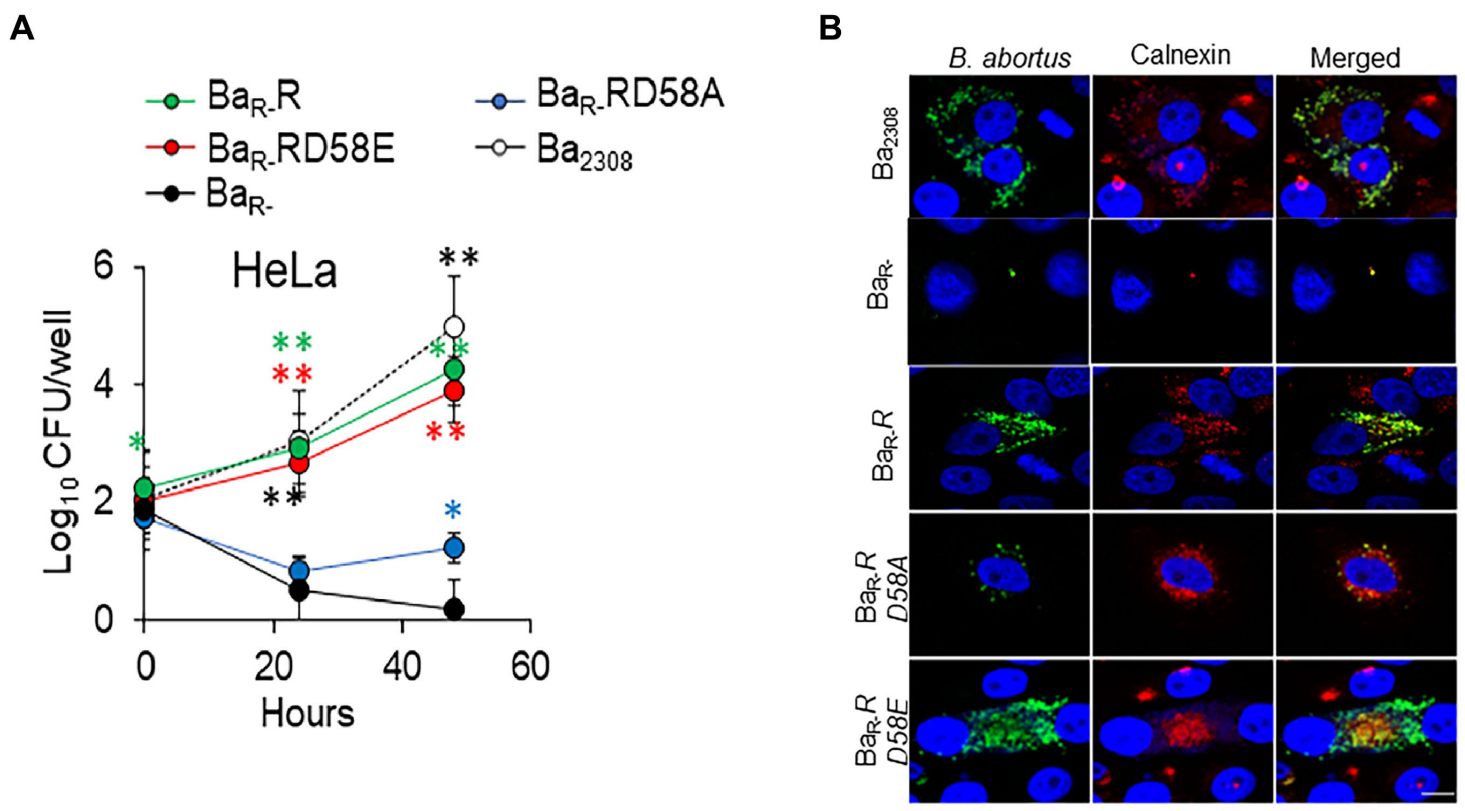
BvrR activation promotes *B. abortus* intracellular replication *ex vivo* at the endoplasmic reticulum. **(A)** HeLa epithelial cells were infected with Ba_2308_ (white circles), Ba_R-_ (black circles), Ba_R-_RD58E (red circles), Ba_R-_R (green circles), and Ba_R-_RD58A (blue circles) grown to mid-exponential growth phase using a MOI of 500. At the indicated times, cells were lysed, and intracellular bacteria were determined by plate counting. Means and standard deviations are shown; *n* = 3 independent experiments. Statistical significance was calculated by ANOVA and Tukey’s multiple-comparison test (*p < 0.05, **p < 0.005 represents Ba_2308_ compared to Ba_R-_*p < 0.05, **p < 0.005 represents BaR-R compared to Ba_R-_, *p < 0.05, **p < 0.005 represents Ba_R-_RD58E compared to Ba_R-_, and *p < 0.05, **p < 0.005 represents BaR-RD58A compared to BaR-). **(B)** HeLa cells were infected using MOI of 500 with the indicated *B. abortus* strains as in **(A)**. At 48 h post-infection, cells were extensively washed, fixed, permeabilized, and intracellular bacteria detected with a mouse-anti *Brucella* antibody and a goat anti-mouse IgG Alexa Fluor Plus 488 conjugate (green). The ER was stained using rabbit anti-calnexin antibodies and a goat anti-Rabbit IgG Alexa Fluor Plus 594 (red). Cells were visualized by confocal microscopy. Scale bars, 10 μm. Data are representative of three independent experiments.

To determine the impact of the BvrR phosphorylation on *B. abortus* intracellular survival, we infected HeLa cells and estimated the intracellular replication of the different *B. abortus* strains. Ba_R-_R and Ba_R-_RD58E showed similar replication to Ba_2308_. These strains showed significantly higher bacterial counts than the Ba_R-_ with more than a 2-log difference in CFU counts at 24 and 48 h post-infection (Figure 3A). Ba_R-_RD58A showed an impaired ability to multiply intracellularly. However, this strain was recovered at significantly higher levels than Ba_R-_ at 48 h indicating partial rescue of the replication defect by the dominant negative version of BvrR (Figure 3A). We then evaluated the multiplication of bacteria within their replicative compartment at 48 h post-infection by colocalization with the ER-marker calnexin. Immunolabeled Ba_2308_, Ba_R-_R, and Ba_R-_RD58E reached high intracellular numbers (250 ± 86, 250 ± 38 and 187 ± 48 bacteria/infected cell respectively) in approximately 1% of cells. In contrast, cells infected with Ba_R-_ showed no replication, with 1 bacterium per infected cell in less than 0.01% of cells, indicative of a substantial replication defect. Ba_R-_RD58A showed an intermediate phenotype with an average of 5 ± 3 bacteria/cell in approximately 0.1% of cells that colocalized with calnexin, reinforcing the conclusion of a partial rescue exerted by the dominant negative version of BvrR (Figure 3B).

### 3.2. Differential impact of BvrR phosphorylation on the expression of genes regulated by BvrRS

**Figure 4:**
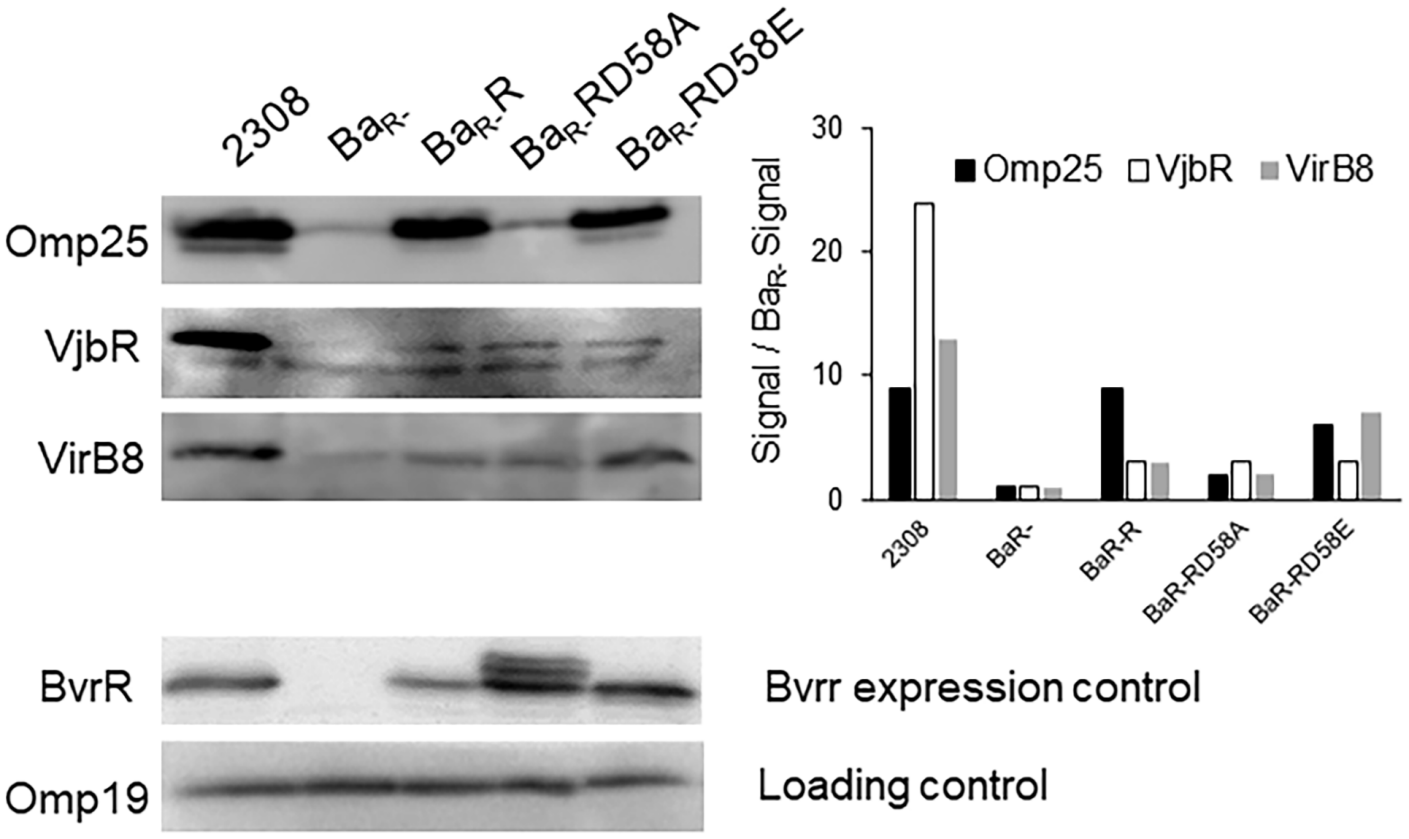
Effect of BvrR phosphorylation on expression of proteins controlled by the TCS BvrRS. Ba_2308_, Ba_R-_, Ba_R-_RD58E, Ba_R-_R, and Ba_R-_RD58A were grown to mid-exponential growth phase in TSB, and aliquots were collected. Equal amounts (20 μg) of whole-bacterium lysates were then separated by 12.5% SDS-PAGE, transferred to PVDF membranes, and probed with anti-BvrR, anti-Omp25, anti VirB8, and anti-VjbR. Data are representative of three independent experiments.

Direct transcriptional control of BvrRS on the *virB* operon, *omp25*, and *vjbR* has previously been reported (Manterola et al., 2007; Martínez-Núñez et al., 2010; Viadas et al., 2010). Here, we sought to determine how the different BvrR activation states affected the abundance of the corresponding proteins by Western blot. BvrR was detectable in Ba_2308_ and BvrR variants but not in the Ba_R-_ strain (Figure 4). The Omp25 levels have a robust signal in Ba_2308_, Ba_R-_R and Ba_R-_RD58E and negligible detection in Ba_R-_ and Ba_R-_ RD58A (Figure 4). In contrast, the levels of VjbR and VirB8 followed a different dynamic. Both proteins were nearly absent in Ba_R-_ as expected for a molecule dependent on BvrRS for its expression (Martínez-Núñez et al., 2010; Viadas et al., 2010; Figure 4). However, the expression of both VjbR and VirB8 was at least partially reconstituted in the three strains harboring the BvrR variants. The level of VjbR was increased three times in Ba_R-_R and Ba_R-_RD58E and Ba_R-_RD58A whereas the level of VirB8 was increased by a factor of 3, 7 and 2, respectively, when compared to Ba_R-_ (Figure 4). Despite of this partial complementation, the levels of these proteins did not achieve the expression seen in the wild type strain as was the case for Omp25. This indicates that even the dominant negative version positively impacts *vjbR* and *virB* expression.

**Figure 5:**
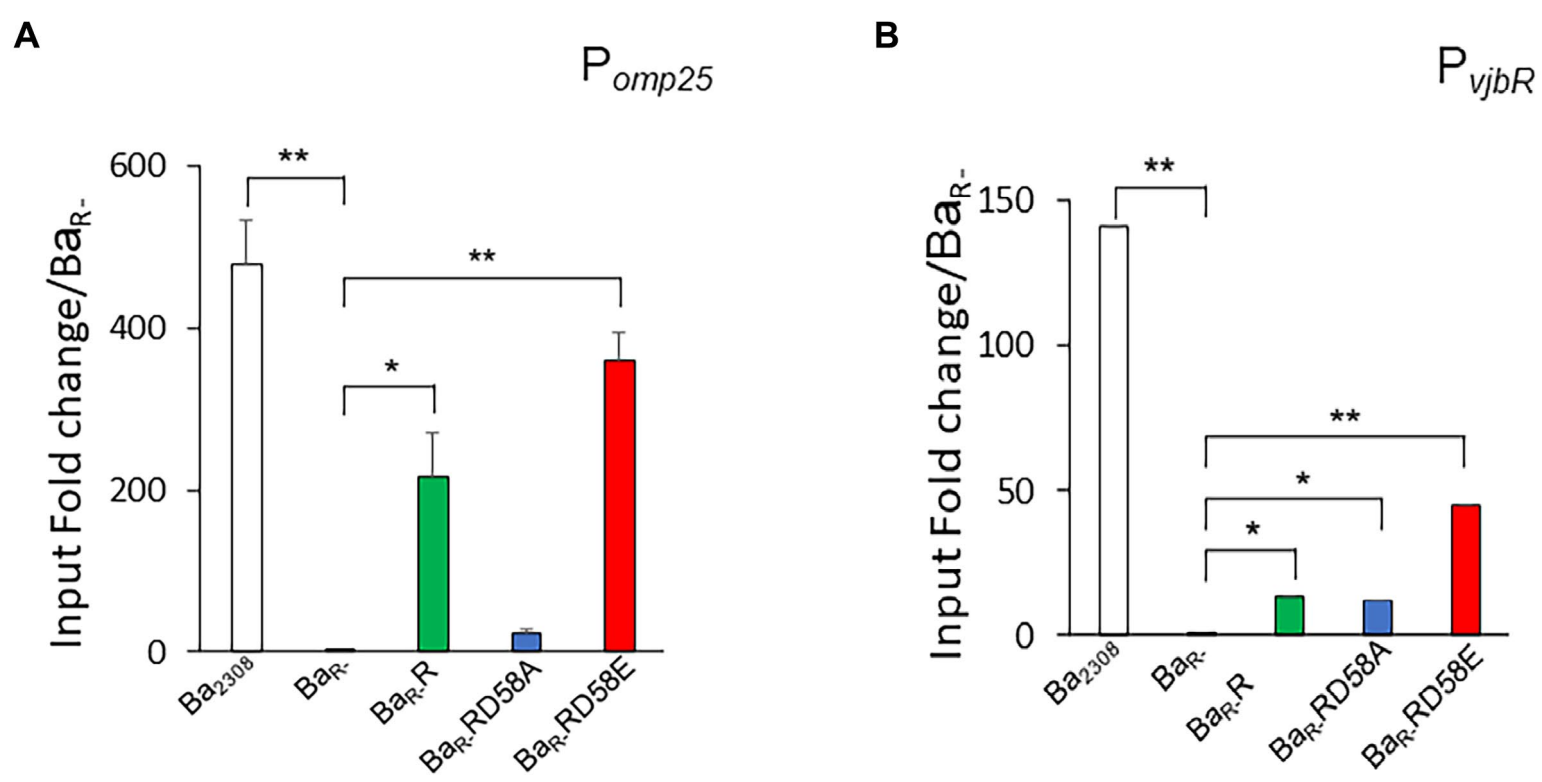
Effect of BvrR phosphorylation on binding to promoters of genes controlled by the TCS BvrRS. *Ba_2308_* (white bars), *Ba_R-_* (black bars), Ba_R-_RD58E (red bars), Ba_R-_R (green bars), and Ba_R-_RD58A (blue bars) were grown in TSB at 37°C to mid-exponential growth phase. Then, bacteria were fixed with formaldehyde (1%), lysed by sonication, and centrifuged. For each sample, an aliquot was taken as a control of total DNA before immunoprecipitation and referred to as total DNA. Then, magnetic beads preloaded with BvrR-antibodies were incubated with the bacteria extracts for 1 h at room temperature. The reaction mixture was extensively washed, and the immunoprecipitated was eluted and neutralized. The cross-linking of the immunoprecipitated was reversed by incubating at 65°C overnight. Then, the DNA was purified, and P*_Omp25_* **(A)**, and P*_vjbR_* **(B)** were amplified by quantitative real-time PCR. Relative quantification by a standard curve is shown. Data were presented as a percentage of precipitated DNA (IP)/total DNA (IN) relative to precipitated DNA in *Ba_R-_*. Means and standard deviations are shown; *n* = 3 independent experiments. Statistical significance was calculated by ANOVA and Tukey’s multiple-comparison test (*p < 0.05 and **p < 0.005 compared to *Ba_R-_*).

We then assessed the binding of the different BvrR protein variants to the promoters of *omp25* (P*omp25*) and *vjb*R (P*vjbR*) using a chromatin immunoprecipitation assay on the different strains grown to mid-exponential phase. The interaction with P*omp25* followed an expected pattern for a gene positively regulated by phosphorylation of the response regulator, with BvrR and BvrRD58E, but not BvrRD58A, binding significantly to the promoter. The interaction of the different variants with P*vjbR* showed a different pattern with the three protein versions, BvrR, BvrRD58E and BvrRD58A showing a significant binding to this promoter (Figure 5).

### 3.3. A global transcriptomics analysis reveals that a subset of the BvrR regulon is controlled by unphosphorylated BvrR

**Figure 6:**
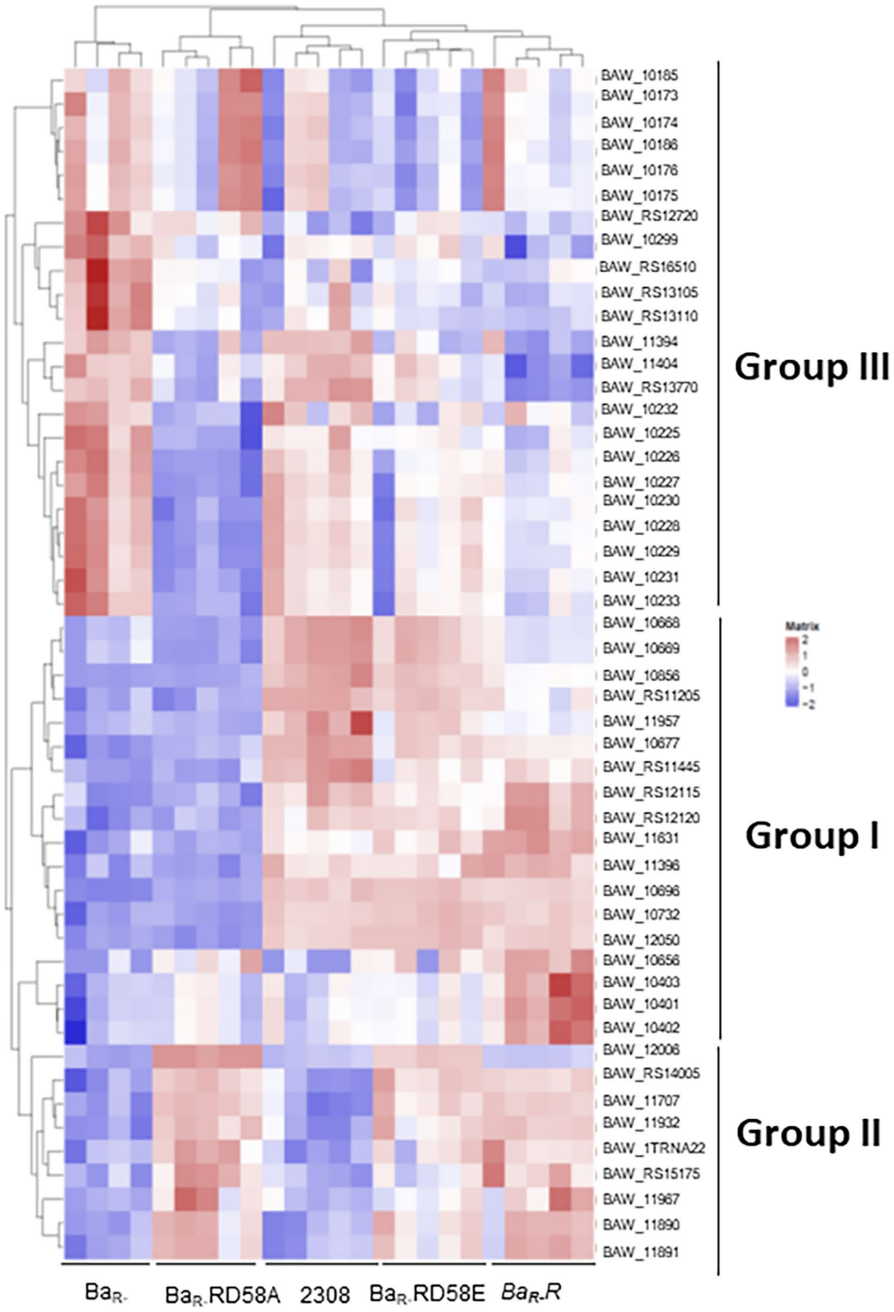
Effect of BvrR phosphorylation on the gene expression of *B. abortus* Ba_2308_, Ba_R-_, Ba_R-_RD58E, Ba_R-_R, and Ba_R-_RD58A were grown in TSB to mid-exponential growth phase. Samples were lysed with TE buffer, pH 8.0 with 1 mg/mL lysozyme, and RNA was then isolated. RNA library was prepared by Illumina TruSeq mRNA Sample Prep kit. Indexed libraries were then submitted to an Illumina NovaSeq and the paired-end (2 × 151 bp) (~40 M reads per sample ~6G/sample) sequencing was performed by the Macrogen Incorporated. Reads were aligned on the *B. abortus genome* (*Brucella abortus* strain Winsconsin from GenBank using bowtie2). Gene expression profiles were analyzed using the R software and several Bioconductor packages. Heatmaps were generated with R, using genes with differential expression (|log2FC| > = 3.5) for at least one pairwise comparison. Each column represents a bacterial sample, and each row represents a gene. The list of genes corresponds to those 54 significantly differentially expressed among at least two bacterial strains (|log2FC| > = 3.5). Genes with high expression (many RNA reads) are shown in red, while genes with low expression are shown in blue. Hierarchical clustering using the Ward.2 method was applied to both bacterial samples and genes so that samples having similar gene expression profiles are clustered together, and so are genes having similar expression profiles.

To determine the impact of the BvrR activation on global gene expression, we performed a transcriptomic analysis by RNA-seq of the strains described above grown in TSB to mid-exponential growth phase. Genes with a differential expression higher than log_2_ = 3.5 between the strains were selected to generate a heatmap. The strains clustered according to the activation state of BvrR, with Ba_R-_ and Ba_R-_RD58A appearing on one side of the heatmap while Ba_2308_, Ba_R-_R and Ba_R-_RD58E clustering on the other side (Figure 6). In depth analysis of the expression level of different groups of genes defined several transcription patterns. Group I genes showed an expression pattern intimately linked to the activation state of BvrR with low levels in Ba_R-_ and Ba_R-_D58A and high levels in Ba_2308_, Ba_R-_R and Ba_R-_D58E (Figure 6). This group included genes previously shown to be positively regulated by BvrRS such as *omp25* and *exoR* (Manterola et al., 2007; Viadas et al., 2010; Rivas-Solano et al., 2022). There is also presence of genes encoding for membrane proteins such as ABC-transporters and lipoproteins confirming the relevance of BvrRS in the homeostasis of the membrane. Group II genes showed an increased expression dependent on BvrR expression in trans regardless of the activation state (Figure 6). This group included *bvrR* itself for obvious reasons. Group III genes seem to be down regulated by BvrR expression regardless of the activation state of the response regulator (Figure 6). Among these, stands the presence of genes encoding for membrane and periplasmic proteins dedicated to oxidoreductase reactions, solute binding and transport including the ABC-transport family previously reported to be increased in BvrRS-deficient mutants (Lamontagne et al., 2007; Viadas et al., 2010). In the transcriptomic analysis a significant difference was found in the level of *vjbR* and *virB8* between Ba_2308_ and Ba_R-_ in agreement with previous studies (Martínez-Núñez et al., 2010; Viadas et al., 2010). This difference was, however, below the threshold selected for the generation of the heatmap.

## 4. Discussion

To get an insight on the regulatory strategies followed by BvrRS, we complemented a *bvrR-* mutant *in trans* with the wild type *bvrR* gene and with two mutated variants of *bvr*R. The first mutation (BvrRD58A) conferred a dominant-negative phenotype that mimics the unphosphorylated BvrR protein. On the other hand, the BvrRD58E mutation behaves as a dominant positive phenotype, mimicking a state of activation by phosphorylation. Even if maximal BvrR phosphorylation is achieved intracellularly, we used strains grown *in vitro* to mid exponential phase due to yield limitations of *ex vivo* recovery of bacteria prohibiting phenotype determination under these conditions.

**Figure 7:**
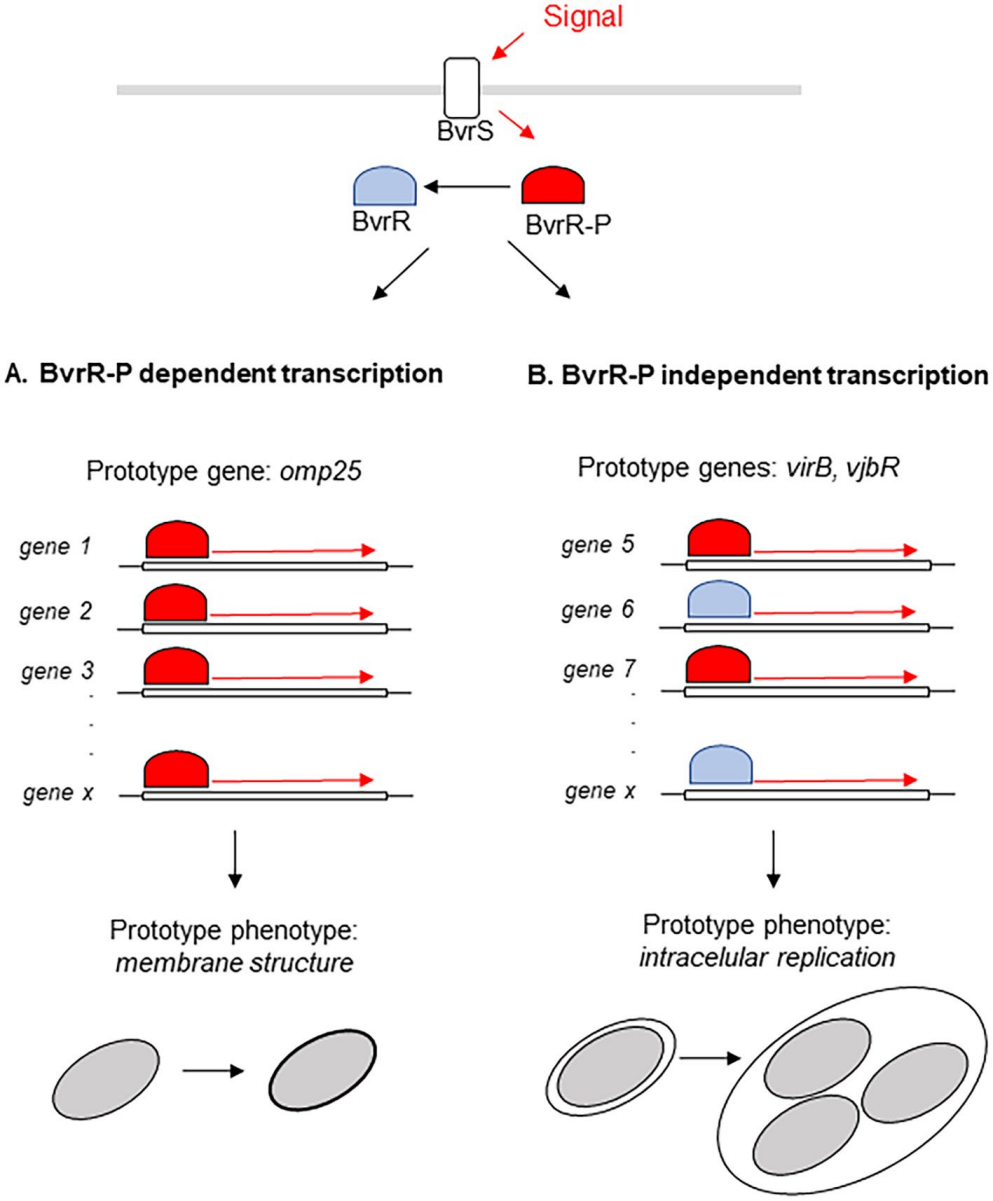
Model of the two types of regulation exerted by BvrR. BvrR/BvrS exerts two types of transcriptional regulation, one fully dependent on BvrR phosphorylation **(A)** and the other allowing participation of un phosphorylated BvrR and with probable participation of additional molecules **(B)**.

The TCS BvrRS plays a role as master regulator in diverse processes ranging from metabolism to virulence control and as consequence mutants defective in this system present defects in several phenotypes (Sola-Landa et al., 1998; Guzmán-Verri et al., 2001; Manterola et al., 2005, 2007). In this work we determined that complementation of a *bvrR*- mutant with *bvrR*, *bvrRD58E* or *bvrRD58A* restored several of those phenotypes to different extents, defining two patterns of regulation mediated by BvrRS (Figure 7). The first pattern, related to membrane integrity, is represented by polymyxin resistance at the phenotypic level and at the transcriptional level by the membrane protein Omp25. In this pattern, both the phenotype and the expression level are fully restored on the wild-type and the dominant positive versions of BvrR whereas the strain complemented with the dominant negative version behaves identical to the *bvrR-* mutant (Figure 7A). The second pattern, related to virulence, is represented at the phenotype level by intracellular replication and at the transcriptional level by VjbR and VirB expression. In this case, not only the wild type and the dominant positive versions of BvrR restore the defective phenotypes but also the dominant negative version confers at least partial complementation. In regard of intracellular replication, the strain harboring the dominant negative mutant achieves significantly higher numbers of intracellular bacteria than the *bvrR-* strain which, furthermore, can reach the ER. Consistent with this behavior, the expression of VjbR and VirB, essential for intracellular survival, are also partially restored in the dominant negative mutant (Figure 7B).

The existence of two different patterns of regulation exerted by BvrR are likely related to the affinity of the phosphorylated and unphosphorylated variants of the response regulator to the corresponding promoters. One would speculate that only phosphorylated BvrR would have sufficient affinity to interact with promoters of genes whose prototype is *omp25*. On the other hand, promoters of genes, represented by *vjbR* and *virB*, would present some affinity for the unphosphorylated isoform of BvrR. This hypothesis received experimental support from our results since (i) BvrRD58A interacted significantly higher than *bvrR-* strain with the *vjbR* promoter, but not with the *omp25* promoter, in the ChiP experiments and (ii) some genes responded to the presence of BvrRD58A in the transcriptomics experiments. The fact that Omp25 is reconstituted to wild type Ba_2308_ levels by the positive variants of BvrR while VjbR and VirB expression is only partially complemented, despite proper binding to the *vjbR* promoter indicated by the ChiP assay, suggest the participation of additional factors in the transcriptional control of the latter proteins and further highlights the existence of different regulatory patterns exerted by BvrR.

Other research groups have also substituted the aspartate residue for alanine or glutamic acid in regulatory proteins belonging to other TCSs and observed various effects of regulatory proteins on different genes (Klose et al., 1993; Mainiero et al., 2010; Little et al., 2018). For example, in *Salmonella typhimurium*, by substituting the aspartate residue of the Nitrogen Regulator Protein (NTR) for alanine, the protein was unable to be activated and induce gene transcription, while substituting glutamate for the aspartate residue the protein achieved a constitutively activated state, which gives it the ability to activate gene transcription (Klose et al., 1993). In *S. aureus* there was a differential expression effect according to the degree of phosphorylation of the regulatory protein SaeR. SaeR phosphorylation was shown to increase the affinity to specific binding sites. Some genes required a high degree of phosphorylation for their regulation, while others were regulated with a low level of phosphorylation of SaeR (Mainiero et al., 2010). In *Pseudomonas aeruginosa*, it was shown that the phosphorylation state of the AlgR regulator differentially regulated the production of pyocyanin and pyoverdin. The AlgR D54A mutant produced low levels of pyoverdin and high levels of pyocyanin compared to the wild-type strain. On the other hand, AlgR D54E produced higher levels of pyoverdin and lower levels of pyocyanin compared to the wild-type strain. Additionally, the AlgR D54A strain showed an attenuation in its replication *in vivo* in mice models (Little et al., 2018). Comparison of the global transcriptional profile of AlgR D54E, AlgR D54A, and the wild-type strain revealed that: (i) gene expression was similar in the wild-type and the AlgR D54E strains, except for five genes, (ii) eight genes belonging to the AlgR regulon were similarly controlled in the algRD54E and algRD54A strains (iii) and 25 genes involved in iron metabolism or its acquisition were differentially expressed between the strains AlgR D54E, AlgR D54A. These data agree with a differential regulation of genes according to the state of phosphorylation of the response regulator. Finally, in *Salmonella enterica*, phosphorylation of the response regulator SsrB is not required to promote biofilm formation. Furthermore, a phosphoablative version of SsrB still binds to the promoter region of *csgD* encoding the master regulator of biofilm formation (Desai et al., 2016).

Our results validate these mutants as a tool to study how BvrR activation affects its regulatory function and gave us a landscape of two regulatory patterns defined by BvrR. These findings serve as a working model for understanding how the response regulators of two-component systems control gene expression.

## Data availability statement

The datasets presented in this study can be found in online repositories. The names of the repository/repositories and accession number(s) can be found at: https://zenodo.org/, 10.5281/zenodo.7548947.

## Author contributions

PA-S generated data and analyzed *in vitro* growth curves, endoplasmic reticulum colocalization analyzes, RNA extraction and analysis, and analyses of the others assays. JM-T and SZ-J generated data and analyzed polymyxin resistance and protein expression Western Blots. NP did RNA-seq mapping and quantification and RNA-seq statistical analyses. GC generated data and analyzed BvrR binding to promoters of genes DNA immunoprecipitation. CC-D analyzed BvrR binding to promoters of genes DNA immunoprecipitation. PA-S and EC-O wrote the first draft of the manuscript. EC-O designed and supervised the study with input from all other authors. PA-S, JM-T, NP, JP, JP-C, CG-V, AZ, CC-D, EM, and EC-O contributed to designed assays, analyzed data, manuscript revision, read, and approved the submitted version. All authors contributed to the article and approved the submitted version.

## Funding

This work was partially supported by FS-CONARE of Costa Rica Grant 803-C2-651 (www.conare.ac.cr), and the Grant 803-C0-456 from Vicerrectoría de Investigación of the University of Costa Rica (www.vinv.ucr.ac.cr). This work was developed within the framework of the agreement between Institute Pasteur, Paris and the University of Costa Rica. Fellowship support for PA-S from SEP-CONARE of the University of Costa Rica for the internship at Institute Pasteur are gratefully acknowledged.

## Conflict of interest

The authors declare that the research was conducted in the absence of any commercial or financial relationships that could be construed as a potential conflict of interest.

## Publisher’s note

All claims expressed in this article are solely those of the authors and do not necessarily represent those of their affiliated organizations, or those of the publisher, the editors and the reviewers. Any product that may be evaluated in this article, or claim that may be made by its manufacturer, is not guaranteed or endorsed by the publisher.
